# A Novel Synergistic Carvacrol–Graphene Oxide–Chitosan Nanocomposite for Effective Root Canal Disinfection

**DOI:** 10.1155/ijod/4667597

**Published:** 2026-07-10

**Authors:** Maryam Monajati, Negin Firouzi, Fahime Alimardani, Mobarakeh Nazari, Pouria Rahnama, Razieh Hoseinzadeh, Mohammad Zare Dorniani, Bahar Asheghi

**Affiliations:** ^1^ Center for Nanotechnology in Drug Delivery, Shiraz University of Medical Sciences, Shiraz, Iran, sums.ac.ir; ^2^ Department of Pharmaceutical Nanotechnology, School of Pharmacy, Shiraz University of Medical Sciences, Shiraz, Iran, sums.ac.ir; ^3^ Department of Endodontics, School of Dentistry, Shiraz University of Medical Sciences, Shiraz, Iran, sums.ac.ir; ^4^ School of Dentistry, Shiraz University of Medical Sciences, Shiraz, Iran, sums.ac.ir

**Keywords:** carvacrol, chitosan, graphene oxide, nanocomposite, root canal disinfection

## Abstract

**Purpose:**

Persistent microorganisms such as *Enterococcus faecalis* and *Candida albicans* remain within complex root canal microanatomy and biofilms, compromising disinfection and leading to endodontic failure. This study aimed to develop and characterize a carvacrol (CV)–graphene oxide (GO)–chitosan (CV@GO–CS) nanocomposite with potential application in root canal disinfection.

**Materials/Methods:**

The CV@GO–CS nanocomposite was synthesized and evaluated for physicochemical properties, encapsulation efficiency (EE%), and release kinetics. Cytocompatibility was assessed using L929 fibroblast viability assays, and cell migration was evaluated by scratch tests. Antimicrobial efficacy was determined against *E. faecalis* and *C. albicans* through MIC/MBC and MIC/MFC measurements and checkerboard synergy assays.

**Results:**

CV@GO–CS exhibited a hydrodynamic diameter of ~190 nm (polydispersity index [PDI] 0.24) and a zeta potential of + 22.3 mV. EE% and loading capacity (LC%) were 88.6% ± 3.6% and 46.9% ± 0.9%, respectively. CV release followed a biphasic profile, with ~45% released at 24 h and ~82% at 96 h. At a concentration of 50 µg/mL—which exceeds the determined minimum bactericidal/fungicidal concentrations—L929 fibroblast viability exceeded 95%, and wound closure was significantly enhanced compared with controls. Antimicrobial testing showed MIC/MBC values of 25 μg/mL against *E. faecalis* and MIC/MFC values of complete inhibition of *C. albicans* at 10 μg/mL.

**Conclusions:**

The CV@GO–CS nanocomposite demonstrated favorable physicochemical characteristics, high cytocompatibility, and strong antimicrobial activity against key endodontic pathogens. These findings support its potential as a sustainable, biocompatible platform for sustained‐release root canal disinfectants or intracanal medicaments; however, efficacy against endodontic biofilms remains to be established, and further preclinical evaluation in biofilm and ex vivo models is warranted.

## 1. Introduction

An essential aspect of endodontic therapy is the effective removal of microorganisms and their byproducts from the root canal system [1]. However, the complex anatomy of root canals and the persistence of resistant microorganisms make complete disinfection a major challenge. Conventional procedures—such as mechanical cleaning and shaping, irrigation, and intracanal medicaments—often fail to eradicate microbes deeply embedded within dentinal tubules or biofilms [2, 3]. Bacteria residing within biofilms exhibit significantly stronger resistance to treatment, forming mixed communities, especially involving *Enterococcus faecalis*, in the interradicular area, which contributes to persistent infections [4].

Although sodium hypochlorite (1%–5%) is considered the gold standard for root canal disinfection, its negative effects—such as unpleasant smell and taste, collagen dissolution in dentin, irritation of soft and hard tissues, and formation of toxic chlorine byproducts—have driven the search for safer and more biocompatible alternatives [5, 6].

Over the years, various nanoparticles have been synthesized and investigated as root canal disinfectants to fight resistant biofilm species [7]. For example, Abbaszadegan et al. [8] reported that chlorhexidine loaded onto positively charged silver nanoparticles had stronger antimicrobial activity against *E. faecalis* than either material alone. Similarly, Vasiliev et al. [9] demonstrated that a combination of silver and copper nanoparticles produced an antibacterial effect up to six times greater than the sum of their individual.

Among different nanomaterials, graphene oxide (GO) nanoparticles (nGO) have gained attention due to their exceptional physicochemical properties, including a large surface‐to‐volume ratio (>200 m^2^/g) and abundant oxygen‐containing functional groups such as hydroxyl, epoxy, and carboxyl groups [10]. These features enhance their dispersibility and reactivity, making them advantageous for biomedical applications, including drug delivery, biosensing, and tissue engineering. Furthermore, the π–π stacking interactions and hydrogen bonding facilitate the efficient loading of bioactive molecules, rendering it a promising scaffold for the construction of multifunctional nanocomposites. This carbon‐based nanomaterial has also demonstrated inherent antibacterial activity, both independently and in combination with other antimicrobial agents [11]. Mejias Carpio et al. [12] demonstrated that functionalization of GO with N‐(trimethoxysilylpropyl)ethylenediamine triacetic acid enhanced its antimicrobial activity while reducing cytotoxicity compared to GO alone. Also, Jouhar et al. [13] reported that methylene blue conjugation to reduced GO significantly increased antifungal and antimicrobial activity.

Chitosan (CS), a natural cationic polysaccharide derived from chitin deacetylation, is widely recognized for its biocompatibility, biodegradability, and notable antimicrobial properties [10]. The positively charged amino groups (pKa ≈ 6.5) interact electrostatically with negatively charged microbial cell surfaces, leading to membrane destabilization and cell lysis. These electrostatic interactions also promote adhesion to biological tissues, making CS an excellent carrier material for biomedical and dental applications [14]. Kishen et al. [15] were among the first to study CS nanoparticles (CS‐NPs) for root canal disinfection, demonstrating their ability to penetrate the complex root canal anatomy and maintain prolonged antibacterial activity for up to 3 months.

Carvacrol (CV), a monoterpenoid phenol and the principal bioactive compound of oregano and thyme essential oils, exhibits potent antimicrobial, antioxidant, and anti‐inflammatory properties [16–18]. Several studies have investigated its use in combination with CS‐NPs, demonstrating enhanced antifungal and antibacterial activities. For instance, Vitali et al. [14] demonstrated that CV‐loaded CS‐NPs exhibited stronger antifungal effects on *Candida albicans* than CV alone, while Akhlaq et al. [19] showed synergistic antimicrobial effects against resistant bacteria.

Although earlier studies demonstrated that combining chitosan and CV improves antimicrobial activity, no research has yet explored the integration of this combination onto nGO. Therefore, the present study aims to develop a novel GO‐based nanocomposite (CV@GO–CS) and to assess its cytotoxicity as well as its antibacterial and antifungal efficacy against *Enterococcus faecalis* and *Candida albicans* under in vitro conditions.

## 2. Materials and Methods

### 2.1. Materials

N‐hydroxysuccinimide (NHS), 1‐ethyl‐3‐(3‐dimethylaminopropyl) carbodiimide hydrochloride (EDC·HCl), CS (MW 100,000–300,000; degree of deacetylation ≥90%), GO, and CV (≥98%) were obtained from Sigma–Aldrich (USA). Tween‐80, acetic acid (glacial), phosphate‐buffered saline (PBS, pH 7.4), cation‐adjusted Mueller–Hinton broth (CAMHB), RPMI‐1640 (with L‐glutamine, without bicarbonate), and 3‐(N‐morpholino)propanesulfonic acid (MOPS) were from Merck (Germany) unless otherwise stated. Ultrapure water (18.2 MΩ cm) was used throughout.

### 2.2. Synthesis of GO–CS Nanocomposite

GO–CS was prepared by amidation of nGO with CS [20]. Briefly, CS (250 mg) was dissolved in 100 mL of 1% (v/v) acetic acid and stirred for 1 h. nGO (200 mg) was dispersed in 25 mL of 0.1 M MES buffer, pH 5.5; EDC·HCl (0.5 g; 2.6 mmol) and NHS (0.6 g; 5.2 mmol) were added, and the suspension was stirred for 3 h at pH 5.5 to activate carboxyl groups. The activated nGO was added dropwise to the CS solution (adjusted to pH ~5.2 with NaOH) and stirred for 12 h at room temperature. The product was collected by centrifugation (~20,000 × g, 10 min), washed twice with 0.1 M acetic acid to remove unreacted CS, rinsed with water, and freeze‐dried.

### 2.3. Preparation of CV‐Loaded GO–CS (CV@GO–CS); Encapsulation Efficiency (EE%) and Loading Capacity (LC%)

Following this, GO–CS (200 mg) was dispersed in 20 mL of 1% (v/v) acetic acid containing 1% (v/v) Tween‐80 [14]. CV was added dropwise at a 1:1 (w/w) GO–CS:CV ratio and gently agitated for 2 h. The product was collected by centrifugation (~20,000 × g, 10 min) and washed with 1% Tween‐80 to remove free CV and then ≥3 times with surfactant‐free water to eliminate residual surfactant. Supernatants were 0.22 µm‐filtered before UV–Vis. Free CV was quantified at 277 nm (UV–Vis spectrophotometer, Cecil CE7250, UK) using a matrix‐matched calibration in 1% Tween‐80 in 1% acetic acid (1–50 µg/mL, *R* ^2^ ≥0.99; 1 cm quartz cuvette) [21]. EE% and LC% were calculated as follows:

EE%=mCV,initial−mCV,freemCV,initial×100100, LC%=mCV,initial−mCV,freemsample×,

where *m*_CV, initial_ is the mass of CV added initially, *m*_CV,free_ is the mass quantified in the pooled supernatants after loading/washes, and *m*_sample_ is the dry mass of the CV@GO–CS sample analyzed. Measurements were performed on three independent batches, each quantified in triplicate.

### 2.4. Physicochemical Characterization

Dynamic light scattering (DLS) was used to measure the hydrodynamic diameter and polydispersity index (PDI) using a HORIBA SZ100‐Z instrument at 25°C. Measurements were performed in filtered deionized water (0.22 µm filter) at a concentration of 0.1 mg/mL with a detection angle of 173° (backscatter). The zeta potential was measured in 1 mM KCl at pH 7.4 and calculated using the Smoluchowski model. For each batch (*n* = 3), three measurements were performed and the results were averaged.

Field‐emission scanning electron microscopy (FE‐SEM) images were obtained using a Mira3 TESCAN microscope (Czech Republic) operated at 15 kV. Samples were sputter‐coated with gold prior to imaging, and representative micrographs are presented with scale bars.

### 2.5. In Vitro Release of CV

The release from CV@GO–CS was assessed by dialysis. Aliquots equivalent to 1 mg nanoparticles in 1 mL were sealed in dialysis tubing (MWCO 12–14 kDa; Spectra/Por) and immersed in PBS (pH 7.4) + 0.5% (v/v) Tween‐80 at 37°C and 100 rpm to maintain sink conditions. At 0.5, 1, 2, 4, 8, 24, 48, 72, and 96 h, *V* = 0.5 mL was withdrawn and replaced with fresh medium. CV was read at 277 nm (Cecil CE7250) using the matrix‐matched calibration; cumulative release at time *t* was corrected for sampling using:

%released t=CtVcell+∑i=1t−1vCiVmCV,loaded×100,

where *C*_*t*_ is the concentration at time *t*, *V*_cell_ is the initial release‐cell volume, *V* is the withdrawn volume at each sampling point that was replaced with fresh medium, *v* = aliquot volume removed each time (e.g., 1 mL), and *m*_CV, loaded_ is the mass of CV loaded in the nanoparticles.

### 2.6. Cell Line Provenance, Culture, and Cytocompatibility (MTT)

L929 mouse fibroblasts (ATCC CCL‐1; RRID:CVCL_0462) were cultured in DMEM (high glucose) + 10% FBS + 1% penicillin/streptomycin at 37°C, 5% CO_2_, ≥95% humidity and used at passages 5–15. For cytocompatibility assessment, cells were seeded at 5000 cells per well in 96‐well plates and allowed to adhere for 24 h. Cytocompatibility was evaluated at a concentration of 50 µg/mL of nGO, 50 µg/mL CS/CV physical mixture, and 50 µg/mL of the CV@GO–CS nanocomposite. Untreated cells served as the negative control. MTT solution (10 µL; 5 mg/mL) was added for 3 h; medium was removed, and formazan was dissolved in 100 µL DMSO; absorbance at 570 nm was read [22]. All experiments were performed in triplicate. Viability was determined as follows:

Cell viability %=absorbance sampleabsorbance control×100.

### 2.7. In Vitro Wound‐Healing (Scratch) Assay

L929 cells were seeded in six‐well plates and grown to ~80% confluence (24 h). A linear scratch was created in L929 monolayers using a sterile 200 µL tip; debris was removed with PBS and fresh DMEM containing 1% FBS, and the test samples (nGO, CS/CV, or CV@GO–CS) were added. Images were captured at 0, 24, and 48 h under identical settings. Scratch width was measured in ImageJ at three positions per field across five fields per well and three wells per group. Percent closure was calculated as follows:

% closure=width0h−widthtwidth0h×100

### 2.8. Microorganisms and Culture Conditions

*Enterococcus faecalis* ATCC 29212 and *Candida albicans* ATCC 10231 were used as representative endodontic pathogens. Bacterial assays used CAMHB; fungal assays used RPMI‐1640 buffered with 0.165 M MOPS to pH 7.0. Working cultures were prepared by two subcultures on Mueller–Hinton agar (bacteria) or Sabouraud dextrose agar (yeasts) prior to testing.

### 2.9. Antimicrobial Susceptibility Testing (MIC, MBC, MFC; CLSI‐Compliant)

Broth microdilution followed CLSI M07 (bacteria) and CLSI M27 (yeasts) [23, 24]. Overnight *E. faecalis* cultures were adjusted to 0.5 McFarland and then diluted to ~5 × 10^5^ CFU/mL in CAMHB. *C. albicans* inocula were ~0.5–2.5 × 10^3^ CFU/mL in RPMI‐MOPS (pH 7.0). Serial two‐fold dilutions of nGO, CS/CV, and CV@GO–CS were prepared in 96‐well plates (final DMSO ≤0.5% when applicable). Controls included medium‐only (sterility) and inoculum‐only (growth). Bacterial MICs were read visually after 18–24 h at 35 ± 2°C as the lowest concentration with no visible growth. Yeast MICs were read visually at 24 and 48 h (35 ± 2°C) using the 50% growth‐reduction endpoint per M27. OD at 600 nm was recorded as a secondary measure; visual endpoints governed MIC calls. For MBC/MFC, 10 µL from wells at and above the MIC were plated onto drug‐free MHA (bacteria) or SDA (yeasts) and incubated (bacteria: 24 h at 35 ± 2°C; yeasts: 24–48 h); MBC/MFC was the lowest concentration producing ≥99.9% (3‐log_10_) reduction versus the starting inoculum.

### 2.10. Synergy Testing (Checkerboard Fractional Inhibitory Concentration Index [FICI])

Two‐dimensional checkerboard microdilution assessed interactions between free CV and blank GO–CS (no CV). Fractional inhibitory concentrations were computed as FIC_A = MIC_A (in combination)/MIC_A(alone) and FIC_B = MIC_B (in combination)/MIC_B(alone); FICI = FIC_A + FIC_B. Interpretation: ≤0.5 synergy, >0.5–1 additive, >1–4 indifferent, and >4 antagonism. Values are reported as the median (IQR) from ≥3 independent experiments.

### 2.11. Statistics

Unless stated otherwise, data are presented as the mean ± SD of independent biological replicates (*n* ≥3); for each experiment, technical replicates were averaged within each biological replicate before analysis. The exact n and test used are reported in each figure legend. Normality (D’Agostino–Pearson) and variance homogeneity (Brown–Forsythe) were assessed; if assumptions were not met, nonparametric alternatives (Mann–Whitney or Kruskal–Wallis with Dunn’s post hoc) were used. Unless specified, tests were two‐sided with *α* = 0.05, and family‐wise error was controlled using Tukey (for ANOVA) or Holm–Šídák (for multiple *t*‐tests), as appropriate. Typical mappings were as follows: MTT—one‐way ANOVA (Dunnett vs. untreated control); scratch closure—two‐way ANOVA (factors: treatment, time; interaction reported); release profiles—nonlinear regression with model comparison by AIC; MIC/MBC/MFC—summarized as medians (IQR) with analyses on log_2_‐transformed MICs or Kruskal–Wallis/Dunn’s when ordinal. FICI values are summarized as the median (IQR) and compared nonparametrically for exploration; categorical synergy calls are descriptive only. Analyses were performed in GraphPad Prism v8.4.3.

## 3. Results

### 3.1. Preparation and Characterization of CV‐Entrapped Nanoparticles

**Figure 1:**
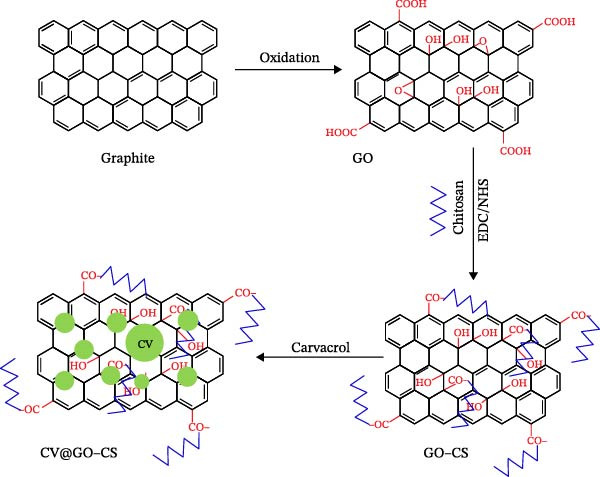
Schematic illustration of the preparation of carvacrol‐loaded graphene oxide–chitosan nanoparticles.

**Figure 2:**
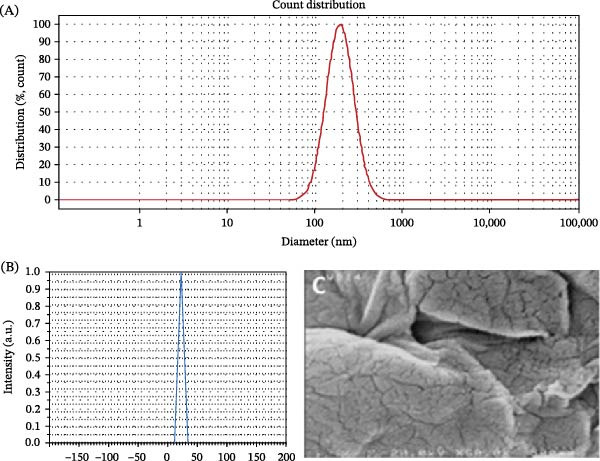
(A) Zeta potential distribution, (B) particle size distribution, and (C) SEM image of GO–CS nanoparticles.

CV@GO–CS nanoparticles were successfully synthesized (Figure 1). The average hydrodynamic diameter of the synthesized nanoparticles was determined using DLS. As shown in Figure 2A, the GO–CS nanoparticles exhibited a unimodal, symmetrical, and narrow size distribution. The average particle size and PDI were 190 nm and 0.24, respectively, indicating a relatively uniform particle population. The zeta potential distribution also showed the same trend and was measured at + 22.3 mV (Figure 2B).

SEM examined the morphology of CV@GO–CS. As shown in Figure 2C, a continuous coating layer with distinct surface texturing and crack‐like patterns was observed. The microstructure exhibited a sheet‐like morphology characterized by multiple surface fractures and a heterogeneous topography, indicating the successful deposition of CS onto the GO substrate.

The loading of CV into GO–CS nanoparticles was evaluated using UV‐Vis spectrophotometric analysis. The EE% and LC% were determined to be 88.6% ± 3.6% and 46.9% ± 0.89%, respectively.

**Figure 3:**
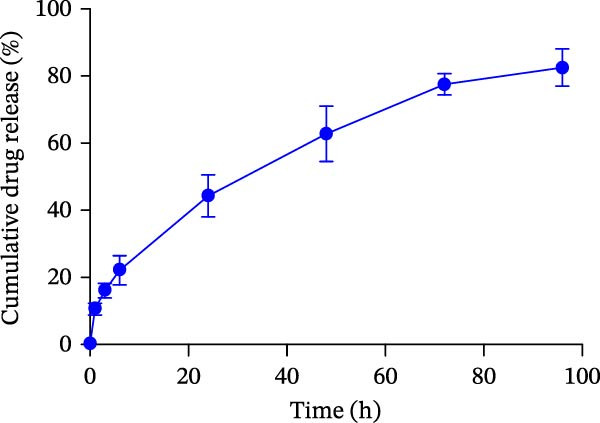
In vitro cumulative release profile of CV from GO–CS nanoparticles in PBS (pH 7.4) + 0.5% (v/v) Tween‐80 at 37°C. Data are mean ± SD (*n* = 3 independent batches).

The in vitro release of CV from the GO–CS nanoparticles exhibited a biphasic pattern (Figure 3). An initial burst release was observed within the first 24 h, reaching ~45% cumulative release. This was followed by a sustained release phase, with 82% cumulative release after 96 h. The release profile indicates controlled drug delivery with sustained release characteristics.

### 3.2. In Vitro Cell Viability

**Figure 4:**
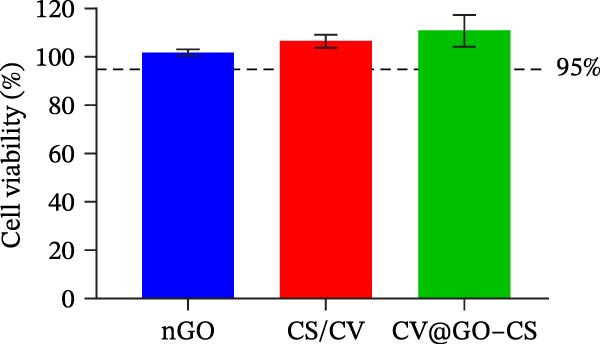
Cell viability of L929 fibroblasts after treatment with nGO, CS/carvacrol physical mixture, and carvacrol‐loaded GO–CS nanoparticles. Data show mean ± SD. One‐way ANOVA with Dunnett’s post hoc test was used for comparisons versus the untreated control; no significant differences were detected (all *p* > 0.05).

A cell viability assay (MTT) was performed to evaluate the cytocompatibility of the synthesized nanoparticles. L929 fibroblasts were treated with 50 µg/mL of nGO, CS/CV, and CV@GO–CS nanoparticles for 72 h. As shown in Figure 4, all formulations had maintained cell viability above 95%, indicating excellent biocompatibility with L929 fibroblasts. There were no statistically significant differences between the treatment groups and control (*p* > 0.05).

### 3.3. In Vitro Wound Healing (Scratch Assay)

**Figure 5:**
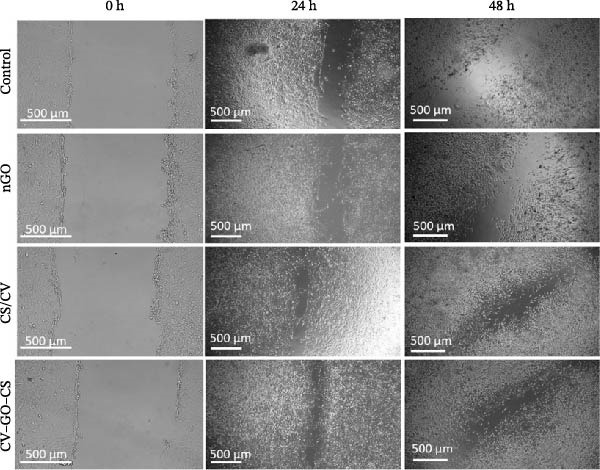
Representative phase contrast micrographs of L929 cells treated with nGO, CS/carvacrol physical mixture, and carvacrol‐loaded GO–CS nanoparticles for 0, 24 h, and 48 h. Untreated wells served as negative controls.

**Figure 6:**
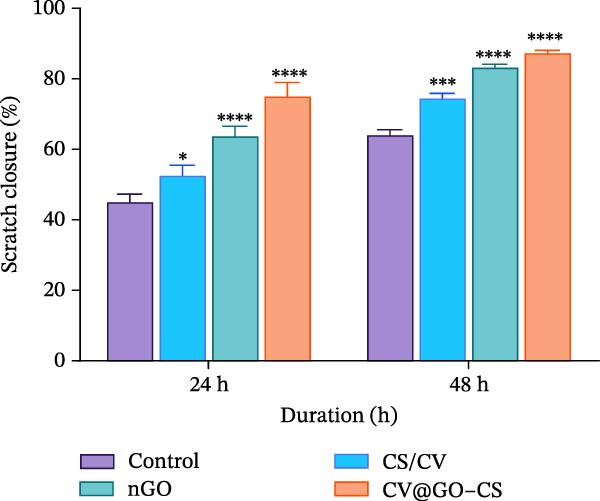
Scratch closure at 24 and 48 h after treatment with nGO, CS/carvacrol physical mixture, and carvacrol‐loaded GO–CS nanoparticles. Control: untreated cells. Data are mean ± SD (*n* = 3 independent experiments; three wells per group). Two‐way ANOVA (factors: treatment, time; interaction reported) with Tukey’s post hoc test;  ^∗^ *p* < 0.05,  ^∗∗∗^ *p* < 0.001,  ^∗∗∗∗^ *p* < 0.0001 vs. control.

An in vitro scratch wound healing assay was carried out to evaluate the effects of GO, CS/CV, and CV@GO–CS nanoparticles on L929 fibroblast migration. As shown in Figures 5 and 6, after 24 h of treatment, all formulations significantly promoted wound closure compared to the control (45.2%), with CV@GO–CS achieving the highest closure (75.2%), followed by CS/CV (63.8%) and GO (52.6%). At 48 h, wound closure further increased, reaching 87.8% for CV@GO–CS and 83.7% for CS/CV, while GO‐treated cells showed 75.0% closure. Notably, CV@GO–CS surpassed other formulations at 24 h, while by 48 h, CS/CV and CV@GO–CS showed comparable performance (*p* > 0.05).

### 3.4. Antibacterial Activity

**Figure 7:**
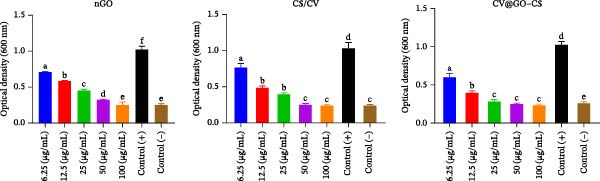
Antibacterial efficacy of nGO, CS/carvacrol physical mixture, and carvacrol‐loaded GO–CS nanoparticles across concentrations of 6.25–100 μg/mL. Wells containing only broth served as the negative control (control−), and wells with microbial suspension but no treatment served as the positive control (control+). Different letters indicate significant differences (Tukey’s test, *p* < 0.05).

The growth inhibitory effects of nGO, CS/CV, and CV@GO–CS nanoparticles were evaluated against *Enterococcus faecalis*. All formulations exhibited dose‐dependent antibacterial activity (Figure 7). For nGO, the MIC was determined to be 100 μg/mL, at which the optical density closely matched that of the sterile control. Lower concentrations showed reduced efficacy, with 6.25 μg/mL retaining ~70% of the turbidity observed in the untreated control. While no significant differences were observed between nGO and CS/CV at lower concentrations (6.25 and 25 μg/mL; *p* > 0.05), CS/CV demonstrated overall enhanced antibacterial activity, with an MIC of 50 μg/mL. The most potent antibacterial effect was achieved with CV@GO–CS, which exhibited an MIC of 25 μg/mL and significantly outperformed both nGO and CS/CV even at concentrations of 6.25–25 μg/mL (*p* < 0.05).

**Figure 8:**
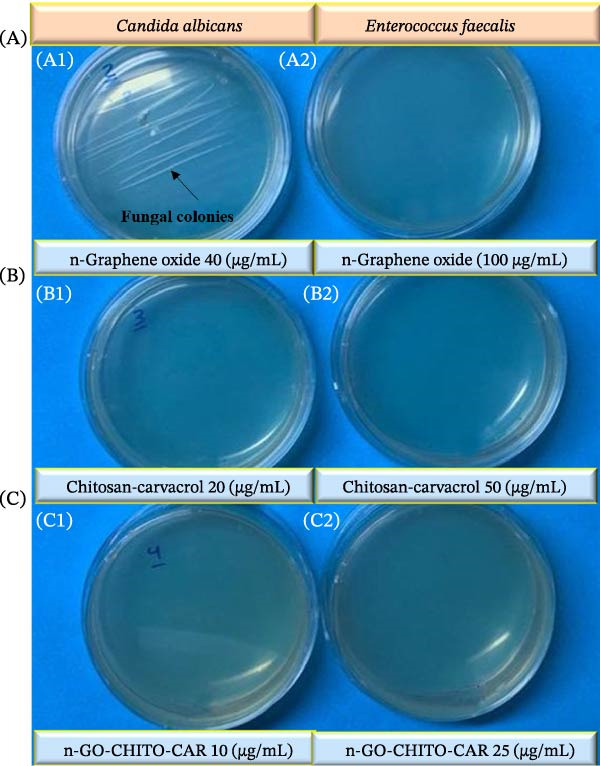
In vitro antimicrobial assay of nanomaterial‐based formulations. (A2, B2, C2) Correspond to *E. faecalis*; (A1, B1, C1) to *C. albicans*. Petri dishes illustrate the inhibitory effects of (A–C) nGO (40 µg/mL for *C. albicans*, 100 µg/mL for *E. faecalis*), CS/CV (20 µg/mL for *C. albicans*, 50 µg/mL for *E. faecalis*), and CV@GO–CS (10 µg/mL for *C. albicans*, 25 µg/mL for *E. faecalis*).

The bactericidal activity of the formulations was further evaluated by determining the MBC. The MBC values were found to be 100 μg/mL for nGO, 50 μg/mL for the CS/CV physical mixture, and 25 μg/mL for the CV@GO–CS nanoparticles, indicating stronger bactericidal activity of the nanocomposite. Representative agar plates confirming these bactericidal concentrations are shown in Figure 8, where no bacterial colonies are observed at the respective MBC levels.

Interaction analysis by checkerboard microdilution against *Enterococcus faecalis* showed that the FICI indicated synergy under our predefined threshold (≤0.5).

Similarly, CV@GO–CS nanoparticles exhibited MFC at ~10 µg/mL. The MFC for CS/CV was higher, around 20 µg/mL. In contrast, nGO demonstrated the weakest antifungal effect, with visible fungal colonies even at the highest tested concentration of 40 µg/mL.

### 3.5. Antifungal Activity

**Figure 9:**
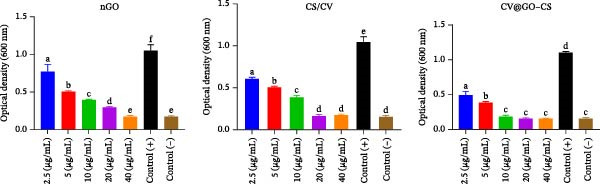
Antifungal efficacy of nGO, CS/carvacrol physical mixture, and carvacrol‐loaded GO–CS nanoparticles across concentrations of 2.5–40 μg/mL. Wells containing only broth served as the negative control (control−), and wells with microbial suspension but no treatment served as the positive control (control+). Different letters indicate significant differences (Tukey’s test, *p* < 0.05).

The antifungal efficacy of different formulations was also investigated against *Candida albicans* (Figure 9). The nGO, CS/CV, and CV@GO–CS nanoparticles demonstrated varying values of the MIC, with concentration‐response relationships observed across all tested formulations. nGO demonstrated fungistatic effects, with complete growth inhibition achieved at 40 μg/mL, corresponding to optical density values equivalent to those of the sterile control. At the lowest tested concentration (2.5 μg/mL), nGO prohibited 25% of fungal growth relative to the untreated control. CS/CV exhibited superior antifungal performance compared to nGO, achieving complete fungal suppression at 20 μg/mL. CV@GO–CS displayed the strongest antifungal activity among all tested formulations, with complete growth inhibition occurring at 10 μg/mL. This nanocomposite consistently outperformed both nGO and CS/CV across the concentration range of 2.5–20 μg/mL (*p* < 0.05), reducing fungal viability to ~50% of the control even at the minimal dose of 2.5 μg/mL, highlighting its exceptional antifungal potency.

Against *Candida albicans*, interaction was evaluated by checkerboard microdilution; the FIC index (FICI) indicated synergy under our predefined threshold (≤0.5).

## 4. Discussion

Given the persistent challenge of eliminating resistant microorganisms from the root canal system, designing nanocomposites with uniform morphology, high colloidal stability, and efficient loading of bioactive compounds is crucial [25]. These attributes are especially decisive for antimicrobial efficacy and delivery safety in complex, confined environments such as the root canals [26, 27]. In the present study, the CV@GO–CS nanocomposite exhibited desirable physicochemical properties, including a uniform particle size distribution and a positive surface charge. The measured parameters indicate favorable uniformity for drug delivery applications. The positive zeta potential also suggests good colloidal stability, likely attributable to positively charged CS at the nanoparticle surface. This feature can enhance adhesion to negatively charged bacterial membranes, thereby increasing the antimicrobial efficacy. SEM analysis confirmed the successful formation of CS–graphene conjugates with continuous surface coverage.

EE% and LC% values indicate the high incorporation of CV into the nanoparticles. This performance likely reflects hydrophobic interactions between the phenolic ring of CV and graphene sheets, together with electrostatic interactions between CS amines and GO carboxyl groups. The biphasic release profile (rapid during the first 24 h, followed by a slower phase to 96 h) indicates a controlled‐release design. The initial burst may arise from CV molecules adsorbed on the nanoparticle surface, whereas the slower phase reflects gradual diffusion from the nanoparticle matrix. This behavior is advantageous for antimicrobial applications because it provides both rapid onset and prolonged effects. A similar pattern was reported by Vitali et al. [14], in which CS‐based nanocarrier systems likewise enabled the gradual and sustained release of phenolic compounds.

Biocompatibility is a key factor for clinical applications. In the present study, the MTT assay showed that treating L929 fibroblasts with GO, the CS/CV physical mixture, or CV@GO–CS did not cause a significant reduction in cell viability, and more than 95% of cells remained viable (*p* > 0.05). Cytocompatibility was evaluated at 50 µg/mL, which remains higher than the determined MBC/MFC values. The high viability observed for all formulations indicates that the effective antimicrobial concentration falls within a cytocompatible range for L929 fibroblasts. A single concentration of 50 µg/mL was chosen for initial cytocompatibility screening because it exceeds the MIC/MBC values determined against both test organisms, in accordance with the ISO 10993‐5 principle that a material should be noncytotoxic at its effective antimicrobial concentration.

However, the scratch assay revealed significant differences between groups: CV@GO–CS achieved 75% wound closure at 24 h and 88% at 48 h, which were significantly higher than those of the GO and CS/CV groups. This finding highlights the strong potential of the nanocomposite to enhance tissue repair in dental applications. The observed advantage can be attributed to several factors. First, CS provides favorable surface adhesion, facilitating fibroblast migration and likely biasing the cellular phenotype toward a reparative state [28, 29]. Second, GO supplies nanoscale topography that upregulates repair‐related genes and helps prevent oxidative stress [30–33]. nGOs have been shown to inhibit LPS‐induced interleukin‐6 (IL‐6) secretion in blood cell cultures [34] [IL‐6]. Moreover, GO‐containing sutures reduced the expression of inflammatory markers in gingival fibroblasts at the suture knot site under LPS stimulation. Third, the presence of CV—owing to its anti‐inflammatory and antioxidant properties—can accelerate fibroblast migration and extracellular matrix formation [35, 36]. In one study, a water‐soluble prodrug of CV alone produced up to 88% wound closure within 24 h, and its combination with hydroxyapatite achieved complete closure (100%) over the same period [36].

Previous studies have reported similar trends; Dinescu et al. [37] showed that a CS–GO hydrogel exhibits higher biocompatibility and cell proliferation than pure CS; similarly, Liao et al. [38] reported that coating GO with CS minimizes its hemolytic effects. Overall, the effects observed in the CV@GO–CS group appear to arise from the synergistic combination of all three components—namely, CS‐mediated cell adhesion, the biological activity of GO, and the anti‐inflammatory properties of CV—rather than from increased cell viability alone.

Pathogenic species such as *Enterococcus faecalis* and *Candida albicans* play major roles in secondary infections and persistent apical periodontitis. These organisms are known for surviving under harsh conditions—including nutrient‐poor, anaerobic environments inside the root canal—and for forming robust biofilms that protect them from antimicrobials and host immune responses [39, 40]. The present study showed that CV@GO–CS exhibits substantial antibacterial and antifungal activities against *E. faecalis* and *C. albicans*. The significant reductions in MIC and MBC/MFC relative to GO and CS/CV indicate a synergistic effect among GO, CS, and CV.

Scientists have found many different mechanisms for graphene to achieve antibacterial activity. First, graphene can physically damage the membrane of the bacteria by coming into direct contact with bacteria using its sharp edge [41]. This severely disrupts the cell membrane, causing cell death. Second, graphene can chemically damage the cell by causing oxidative stress through the production of reactive oxygen species (ROS) [42, 43]. Third, graphene can disrupt the membrane of bacteria through electron transfer. The graphene can act as an electron acceptor by removing electrons from the bacterial membrane and disrupting the membrane integrity, resulting in cell death [44, 45]. When CS interacts with negative charges of cell membranes, amino groups disrupt the membrane structure and induce microbial death [46, 47]. However, CS cannot be dissolved in neutral aqueous solutions or organic solvents, which limits its application in certain fields. In addition, the antibacterial effect has been indicated to be weaker in neutral environments [46]. Therefore, modification of CS to enhance its solubility while maintaining its antibacterial ability may allow it to be applied in a broader range of conditions [48]. CV has a multiple action mechanism based on alteration of the cytoplasmic membrane, interruption of the flow of electrons, interference with active transport, and inhibition of coagulation of the cytoplasmic content and of enzyme activity, and the sustained release of CV permeabilizes the lipid bilayer, leading to intracellular leakage [18]. Nanda et al. [49] used Raman spectroscopy to show that GO disrupts the membrane of *E. faecalis*, causing leakage of adenine and proteins. Martini et al. [50] likewise reported weaker efficacy for GO alone and its enhancement in the presence of bioactive agents. In Eskandari et al. [51], combining GO with double antibiotic paste produced significant antibacterial activity.

For *C. albicans*, CV binds to membrane sterols, disrupting permeability and inducing membrane depolarization [52], whereas CS enhances antifungal effects by inhibiting ergosterol synthesis and compromising the fungal cell wall [53]. In Vitali et al. [14], CV–CS‐NPs showed an MIC of 24 µg/mL, which is consistent with the findings of the present study (MIC = 20 µg/mL). Overall, the CV@GO–CS formulation exhibits superior antifungal and antibacterial activities compared to those of each component.

Effective endodontic disinfection requires the penetration of antimicrobial agents into the complex microanatomy of the root canal, including the dentinal tubules, which typically range from 1 to 3 μm in diameter. The CV@GO–CS nanocomposite synthesized in this study exhibits a hydrodynamic diameter of ~190 nm (PDI 0.24) and a positive zeta potential of + 22.3 mV. These physicochemical properties indicate a high potential for the nanoparticles to diffuse deeply into the dentinal tubules to reach embedded microorganisms. Additionally, the positive surface charge may facilitate favorable electrostatic interactions with the negatively charged dentin walls, potentially prolonging the antimicrobial presence. While the current findings establish the baseline efficacy of the nanocomposite, future ex vivo investigations utilizing confocal laser scanning microscopy (CLSM) on extracted human teeth are required to precisely quantify the extent and depth of its tubular penetration [54–56].

### 4.1. Limitations and Future Directions

According to the results obtained from this in vitro study, however, the primary limitation of this study is the exclusive reliance on planktonic microbial models rather than biofilm‐based systems that more faithfully replicate the physiological state of endodontic infections. Future work should employ static or flow cell biofilm models, as well as infected dentin block or extracted tooth models, to evaluate the antibiofilm and tubular penetration capabilities of CV@GO–CS. Furthermore, while this study compared the nanocomposite against its individual constituents to establish synergy, the absence of direct comparisons with gold standard clinical irrigants such as sodium hypochlorite and chlorhexidine limits the immediate clinical interpretability of the MIC/MBC values. Because such comparisons are most meaningful in biofilm or ex vivo models rather than in simple planktonic assays, future studies must include these comparators under clinically relevant conditions. Also, the cytotoxicity assessment was limited to a single cell line (L929 fibroblasts) at a single concentration. While this represents a standard initial screening approach for biomedical materials, broader biocompatibility profiling—including dose–response curves, testing on dental pulp stem cells or periodontal ligament fibroblasts, and longer exposure times—is necessary to fully establish the safety profile of CV@GO–CS.

## 5. Conclusions

In summary, we synthesized and characterized a novel nanocomposite of CV@GO–CS that exhibited improved antimicrobial and wound healing properties for endodontic applications. The nanocomposite had a uniform nanoscale structure, high drug loading efficiency, and a controlled release profile over 96 h. It also showed potent antimicrobial activity against *Enterococcus faecalis* and *Candida albicans*, which was fourfold improved over GO alone. Furthermore, CV@GO–CS exhibited favorable biocompatibility, enhanced fibroblast migration, and accelerated wound closure. These findings suggest that the synergistic combination of CS, GO, and CV provides a multifunctional platform with antimicrobial and pretherapeutic capabilities. However, efficacy against endodontic biofilms has not yet been determined, and further preclinical evaluation in biofilm and ex vivo models is necessary, and in vivo studies are recommended to evaluate toxicity, pharmacokinetics, and therapeutic efficacy in more complex biological models before clinical translation.

## Author Contributions

**Bahar Asheghi**: conceptualization, methodology, writing – original draft, writing – review and editing, funding acquisition. **Maryam Monajati**: conceptualization, methodology, writing – original draft, funding acquisition. **Negin Firouzi**: conceptualization, investigation. **Pouria Rahnama**: investigation, formal analysis, writing – original draft. **Fahime Alimardani and Mobarakeh Nazari**: investigation, data curation, writing – original draft. **Razieh Hoseinzadeh and Mohammad Zare Dorniani**: writing – original draft.

## Funding

This research was supported by a grant from the Shiraz University of Medical Sciences (Grant 30718).

## Disclosure

All authors have read and approved the final version of the manuscript. Bahar Asheghi had full access to all of the data in this study and takes complete responsibility for the integrity of the data and the accuracy of the data analysis. The sponsors had no role in the design, execution, interpretation, or writing of the study.

## Ethics Statement

This in vitro study did not involve human participants or animals. Ethical approval was obtained from the Ethics Committee of Shiraz University of Medical Sciences (IR.SUMS.DENTAL.REC.1403.043).

## Conflicts of Interest

The authors declare no conflicts of interest.

## Data Availability

The data that support the findings of this study are available from the corresponding author upon reasonable request.

## References

[1] Ng R. , Singh F. , Papamanou D. A. , Song X. , Patel C. , and Holewa C. , Endodontic Photodynamic Therapy Ex Vivo, Journal of Endodontics. (2011) 37, no. 2, 217–222, 10.1016/j.joen.2010.10.008. 21238805 PMC3034089

[2] Komine C. and Tsujimoto Y. , A Small Amount of Singlet Oxygen Generated via Excited Methylene Blue by Photodynamic Therapy Induces the Sterilization of *Enterococcus faecalis* , Journal of Endodontics. (2013) 39, no. 3, 411–414, 10.1016/j.joen.2012.11.051. 23402518

[3] Bottcher D. E. , Sehnem N. T. , Montagner F. , Fatturi Parolo C. C. , and Grecca F. S. , Evaluation of the Effect of *Enterococcus faecalis* Biofilm on the 2% Chlorhexidine Substantivity: An in Vitro Study, Journal of Endodontics. (2015) 41, no. 8, 1364–1370, 10.1016/j.joen.2015.04.016. 26025346

[4] Parolia A. , Kumar H. , Ramamurthy S. , Davamani F. , and Pau A. , Effectiveness of Chitosan-Propolis Nanoparticle Against *Enterococcus faecalis* Biofilms in the Root Canal, BMC Oral Health. (2020) 20, no. 1, 10.1186/s12903-020-01330-0. PMC7690148 33238961

[5] Cai C. , Chen X. , Li Y. , and Jiang Q. , Advances in the Role of Sodium Hypochlorite Irrigant in Chemical Preparation of Root Canal Treatment, Biomed Research International. (2023) 2023, no. 1, 10.1155/2023/8858283, 8858283. 36685672 PMC9859704

[6] Ioannidis K. , Niazi S. , Deb S. , Mannocci F. , Smith D. , and Turner C. , Quantification by SIFT-MS of Volatile Compounds Produced by the Action of Sodium Hypochlorite on a Model System of Infected Root Canal Content, PLoS One. (2018) 13, no. 9, 10.1371/journal.pone.0198649, e0198649. 30199524 PMC6130855

[7] Bassegoda A. , Ivanova K. , Ramon E. , and Tzanov T. , Strategies to Prevent the Occurrence of Resistance Against Antibiotics by Using Advanced Materials, Applied Microbiology and Biotechnology. (2018) 102, no. 5, 2075–2089, 10.1007/s00253-018-8776-0. 29392390

[8] Abbaszadegan A. , Tayebikhorami E. , Gholami A. , Bonyanpour N. , Asheghi B. , and Nikmanesh S. , Synergistic Bactericidal Activity of Chlorhexidine Loaded on Positively Charged Ionic Liquid-Protected Silver Nanoparticles as a Root Canal Disinfectant against *Enterococcus faecalis*: An Ex Vivo Study, Journal of Ionic Liquids. (2024) 4, no. 2, 10.1016/j.jil.2024.100117, 100117.

[9] Vasiliev G. , Kubo A. L. , and Vija H. , et al.Synergistic Antibacterial Effect of Copper and Silver Nanoparticles and Their Mechanism of Action, Scientific Reports. (2023) 13, no. 1, 10.1038/s41598-023-36460-2. PMC10244331 37280318

[10] Capuano N. , Amato A. , Dell’Annunziata F. , Giordano F. , Folliero V. , and Di Spirito F. , Nanoparticles and Their Antibacterial Application in Endodontics, Antibiotics. (2023) 12, no. 12, 10.3390/antibiotics12121690, 1690. 38136724 PMC10740835

[11] Cacaci M. , Martini C. , Guarino C. , Torelli R. , Bugli F. , and Sanguinetti M. , Graphene Oxide Coatings as Tools to Prevent Microbial Biofilm Formation on Medical Device, Yeast Membrane Transport. (2020) 1282, 21–35, 10.1007/5584_2019_434. 31468360

[12] Mejias Carpio I. E. , Mangadlao J. D. , Nguyen H. N. , Advincula R. C. , and Rodrigues D. F. , Graphene Oxide Functionalized With Ethylenediamine Triacetic Acid for Heavy Metal Adsorption and Anti-Microbial Applications, Carbon. (2014) 77, 289–301, 10.1016/j.carbon.2014.05.032.

[13] Jouhar R. , Halim M. S. , Ahmed M. A. , Shah F. , and Quadri S. A. , Synthesis, Characterization, and Application of Methylene Blue Functionalized Reduced Graphene Oxide for Photodynamic Therapy in Root Canal Treatment, Pakistan Journal of Medical Sciences. (2025) 41, no. 2, 519–524, 10.12669/pjms.41.2.11001. 39926673 PMC11803785

[14] Vitali A. , Stringaro A. , Colone M. , Muntiu A. , and Angiolella L. , Antifungal Carvacrol Loaded Chitosan Nanoparticles, Antibiotics. (2021) 11, no. 1, 10.3390/antibiotics11010011. PMC8773451 35052888

[15] Kishen A. , Shi Z. , Shrestha A. , and Neoh K. G. , An Investigation on the Antibacterial and Antibiofilm Efficacy of Cationic Nanoparticulates for Root Canal Disinfection, Journal of Endodontics. (2008) 34, no. 12, 1515–1520, 10.1016/j.joen.2008.08.035. 19026885

[16] Miranda-Cadena K. , Marcos-Arias C. , Mateo E. , Aguirre-Urizar J. M. , Quindos G. , and Eraso E. , In Vitro Activities of Carvacrol, Cinnamaldehyde and Thymol Against Candida Biofilms, Biomedicine & Pharmacotherapy. (2021) 143, 10.1016/j.biopha.2021.112218, 112218. 34649348

[17] El Abed S. , k Ibnsouda S. , Latrache H. , Zineb G. , Mouradi H. , and Remmal A. , Carvacrol and Thymol Components Inhibiting *Pseudomonas aeruginosa* Adherence and Biofilm Formation, African Journal of Microbiology Research. (2011) 5, no. 20, 3229–3232, 10.5897/AJMR11.275.

[18] Fernandez-Babiano I. , Navarro-Perez M. L. , Perez-Giraldo C. , and Fernandez-Calderon M. C. , Antibacterial and Antibiofilm Activity of Carvacrol Against Oral Pathogenic Bacteria, Metabolites. (2022) 12, no. 12, 10.3390/metabo12121255, 1255. 36557293 PMC9785330

[19] Akhlaq A. , Ashraf M. , Ovais Omer M. , and Altaf I. , Synergistic Antibacterial Activity of Carvacrol Loaded Chitosan Nanoparticles With Topoisomerase Inhibitors and Genotoxicity Evaluation, Saudi Journal of Biological Sciences. (2023) 30, no. 9, 10.1016/j.sjbs.2023.103765, 103765. 37609545 PMC10440572

[20] Chowdhuri A. R. , Tripathy S. , Chandra S. , Roy S. , and Sahu S. K. , A ZnO Decorated Chitosan–Graphene Oxide Nanocomposite Shows Significantly Enhanced Antimicrobial Activity With ROS Generation, RSC Advances. (2015) 5, no. 61, 49420–49428, 10.1039/C5RA05393E.

[21] Lin L. , Zhu Y. , Thangaraj B. , Abdel-Samie M. A. , and Cui H. , Improving the Stability of Thyme Essential Oil Solid Liposome by Using β-Cyclodextrin as a Cryoprotectant, Carbohydrate Polymers. (2018) 188, 243–251, 10.1016/j.carbpol.2018.02.010. 29525162

[22] Zielińska-Górska M. , Hotowy A. , Wierzbicki M. , Bałaban J. , Sosnowska M. , and Jaworski S. , Graphene Oxide Nanofilm and the Addition of l-Glutamine Can Promote Development of Embryonic Muscle Cells, Journal of Nanobiotechnology. (2020) 18, no. 1, 10.1186/s12951-020-00636-z. PMC7229609 32414365

[23] Clinical, Laboratory Standards I , Methods for Dilution Antimicrobial Susceptibility Tests for Bacteria That Grow Aerobically, 2024, 12th edition, Clinical and Laboratory Standards Institute.

[24] Clinical, Laboratory Standards I , Reference Method for Broth Dilution Antifungal Susceptibility Testing of Yeasts, 2017, 4th edition, Clinical and Laboratory Standards Institute.

[25] Dr Saneyah A. , Dr Zain H. , Dr Asad F. , s DrBisma , Dr Aiman M. , and Dr Syed Abul F. , The Role of the Microbiome in Endodontic Treatment Failure, International Journal of Pharmacy Research & Technology (IJPRT). (2025) 15, no. 1, 327–329.

[26] Wu B. , Zhou Z. , and Hong X. , et al.Novel Approaches on Root Canal Disinfection Methods Against *E. faecalis* , Journal of Oral Microbiology. (2025) 17, no. 1, 10.1080/20002297.2025.2475947, 2475947. 40066376 PMC11892053

[27] Bazzaz B. S. F. , Fakori M. , Khameneh B. , and Hosseinzadeh H. , Effects of Omeprazole and Caffeine Alone and in Combination With Gentamicin and Ciprofloxacin Against Antibiotic Resistant *Staphylococcus aureus* and *Escherichia coli* Strains, Journal of Pharmacopuncture. (2019) 22, no. 1, 49–54, 10.3831/KPI.2019.22.006. 30989001 PMC6461300

[28] Harugade A. , Sherje A. P. , and Pethe A. , Chitosan: A Review on Properties, Biological Activities and Recent Progress in Biomedical Applications, Reactive and Functional Polymers. (2023) 191, 10.1016/j.reactfunctpolym.2023.105634, 105634.

[29] Asheghi B. , Asadi K. , Gholami A. , Enteghad M. , Sadeghi S. S. , and Firouzi N. , Chitosan Nanogels Enriched With Granulocyte-Macrophage Colony-Stimulating Growth Factor Promote Odontoblastic Differentiation in Human Dental Pulp Stem Cells in Vitro, BMC Oral Health. (2025) 25, no. 1, 10.1186/s12903-025-06185-x. PMC12220100 40597938

[30] Tian X. , Yang Z. , and Duan G. , et al.Graphene Oxide Nanosheets Retard Cellular Migration via Disruption of Actin Cytoskeleton, Small. (2017) 13, no. 3, 10.1002/smll.201602133, 1602133. 27762498

[31] Moradi S. , Hamedi H. , Tonelli A. E. , and King M. W. , Chitosan/Graphene Oxide Composite Films and Their Biomedical and Drug Delivery Applications: A Review, Applied Sciences. (2021) 11, no. 17, 10.3390/app11177776, 7776.

[32] Dacrory S. , D’Amora U. , and Longo A. , et al.Chitosan/Cellulose Nanocrystals/Graphene Oxide Scaffolds as a Potential pH-Responsive Wound Dressing: Tuning Physico-Chemical, Pro-Regenerative and Antimicrobial Properties, International Journal of Biological Macromolecules. (2024) 278, no. Pt 1, 10.1016/j.ijbiomac.2024.134643, 134643. 39128733

[33] Cervero-Varona A. , Canciello A. , and Peserico A. , et al.Graphene Oxide Accelerates TGFbeta-Mediated Epithelial-Mesenchymal Transition and Stimulates Pro-Inflammatory Immune Response in Amniotic Epithelial Cells, Materials Today Bio. (2023) 22, 10.1016/j.mtbio.2023.100758, 100758. PMC10432246 37600353

[34] Lategan K. , Alghadi H. , Bayati M. , de Cortalezzi M. F. , and Pool E. , Effects of Graphene Oxide Nanoparticles on the Immune System Biomarkers Produced by RAW 264.7 and Human Whole Blood Cell Cultures, Nanomaterials. (2018) 8, no. 2, 10.3390/nano8020125. PMC5853756 29495255

[35] Gunal M. Y. , Heper A. O. , and Zaloglu N. , The Effects of Topical Carvacrol Application on Wound Healing Process in Male Rats, Pharmacognosy Journal. (2014) 6, no. 3, 10–13, 10.5530/pj.2014.3.2.

[36] Marinelli L. , Cacciatore I. , and Costantini E. , et al.Wound-Healing Promotion and Anti-Inflammatory Properties of Carvacrol Prodrugs/Hyaluronic Acid Formulations, Pharmaceutics. (2022) 14, no. 7, 10.3390/pharmaceutics14071468, 1468. 35890363 PMC9323613

[37] Dinescu S. , Ionita M. , and Pandele A. M. , et al.In Vitro Cytocompatibility Evaluation of Chitosan/Graphene Oxide 3D Scaffold Composites Designed for Bone Tissue Engineering, Bio-Medical Materials and Engineering. (2014) 24, no. 6, 2249–2256, 10.3233/BME-141037. 25226924

[38] Liao K. H. , Lin Y. S. , Macosko C. W. , and Haynes C. L. , Cytotoxicity of Graphene Oxide and Graphene in Human Erythrocytes and Skin Fibroblasts, ACS Applied Materials & Interfaces. (2011) 3, no. 7, 2607–2615, 10.1021/am200428v. 21650218

[39] Ahmed S. , Jehad Hassan S. , and Gajdhar S. , et al.Prevalence of *Enterococcus faecalis* and *Candida albicans* in Endodontic Retreatment Cases: A Comprehensive Study, The Saudi Dental Journal. (2024) 36, no. 4, 539–545, 10.1016/j.sdentj.2024.01.009. 38690386 PMC11056411

[40] Alberti A. , Corbella S. , Taschieri S. , Francetti L. , Fakhruddin K. S. , and Samaranayake L. P. , Fungal Species in Endodontic Infections: A Systematic Review and Meta-Analysis, PLoS One. (2021) 16, no. 7, 10.1371/journal.pone.0255003, e0255003. 34293029 PMC8297845

[41] Akhavan O. and Ghaderi E. , Toxicity of Graphene and Graphene Oxide Nanowalls Against Bacteria, ACS Nano. (2010) 4, no. 10, 5731–5736, 10.1021/nn101390x. 20925398

[42] Liu S. , Zeng T. H. , and Hofmann M. , et al.Antibacterial Activity of Graphite, Graphite Oxide, Graphene Oxide, and Reduced Graphene Oxide: Membrane and Oxidative Stress, ACS Nano. (2011) 5, no. 9, 6971–6980, 10.1021/nn202451x. 21851105

[43] Dutta T. , Sarkar R. , and Pakhira B. , et al.ROS Generation by Reduced Graphene Oxide (rGO) Induced by Visible Light Showing Antibacterial Activity: Comparison With Graphene Oxide (GO), RSC Advances. (2015) 5, no. 98, 80192–80195, 10.1039/C5RA14061G.

[44] Williams A. G. , Moore E. , Thomas A. , and Johnson J. A. , Graphene-Based Materials in Dental Applications: Antibacterial, Biocompatible, and Bone Regenerative Properties, International Journal of Biomaterials. (2023) 2023, 10.1155/2023/8803283, 8803283. 36819211 PMC9929215

[45] Akhavan O. and Ghaderi E. , Photocatalytic Reduction of Graphene Oxide Nanosheets on TiO_2_ Thin Film for Photoinactivation of Bacteria in Solar Light Irradiation, The Journal of Physical Chemistry C. (2009) 113, no. 47, 20214–20220, 10.1021/jp906325q.

[46] Kong M. , Chen X. G. , Xing K. , and Park H. J. , Antimicrobial Properties of Chitosan and Mode of Action: A State of the Art Review, International Journal of Food Microbiology. (2010) 144, no. 1, 51–63, 10.1016/j.ijfoodmicro.2010.09.012. 20951455

[47] Shariatinia Z. , Pharmaceutical Applications of Chitosan, Advances in Colloid and Interface Science. (2019) 263, 131–194, 10.1016/j.cis.2018.11.008. 30530176

[48] Wang N. , Ji Y. , and Zhu Y. , et al.Antibacterial Effect of Chitosan and Its Derivative on *Enterococcus faecalis* Associated With Endodontic Infection, Experimental and Therapeutic Medicine. (2020) 19, no. 6, 3805–3813, 10.3892/etm.2020.8656. 32346445 PMC7185077

[49] Nanda S. S. , Yi D. K. , and Kim K. , Study of Antibacterial Mechanism of Graphene Oxide Using Raman Spectroscopy, Scientific Reports. (2016) 6, 10.1038/srep28443, 28443. 27324288 PMC4914938

[50] Martini C. , Longo F. , and Castagnola R. , et al.Antimicrobial and Antibiofilm Properties of Graphene Oxide on *Enterococcus faecalis* , Antibiotics. (2020) 9, no. 10, 10.3390/antibiotics9100692. PMC7602102 33066198

[51] Eskandari F. , Abbaszadegan A. , Gholami A. , and Ghahramani Y. , The Antimicrobial Efficacy of Graphene Oxide, Double Antibiotic Paste, and Their Combination Against *Enterococcus faecalis* in Root Canal Treatment, BMC Oral Health. (2023) 23, no. 1, 10.1186/s12903-023-02718-4. PMC9840282 36639767

[52] Niu C. , Wang C. , and Yang Y. , et al.Carvacrol Induces *Candida albicans* Apoptosis Associated With Ca(2+)/Calcineurin Pathway, Frontiers in Cellular and Infection Microbiology. (2020) 192, 10.3389/fcimb.2020.00192. PMC7203418 32426298

[53] Srihawan O. , Panichuttra A. , Lertchirakarn V. , and Matangkasombut O. , Efficacy of Chitosan Root Canal Medicament Against Cross-Kingdom Dual-Species Biofilm of *Candida albicans* and *Enterococcus faecalis* in an in Vitro Root-Canal Model, Odontology. (2025) 113, no. 3, 956–964, 10.1007/s10266-024-01024-x. 39540969

[54] Kesim B. , Burak A. , Üstün Y. , Delikan E. , and Güngör A. , Effect of Chitosan on Sealer Penetration Into the Dentinal Tubules, Nigerian Journal of Clinical Practice. (2018) 21, no. 10, 1284–1290, 10.4103/njcp.njcp_127_18. 30297560

[55] Shrestha A. , Fong S.-W. , Khoo B.-C. , and Kishen A. , Delivery of Antibacterial Nanoparticles Into Dentinal Tubules Using High-Intensity Focused Ultrasound, Journal of Endodontics. (2009) 35, no. 7, 1028–1033, 10.1016/j.joen.2009.04.015. 19567328

[56] Gevkaliuk N. , Martyts I. , Mykhailiuk V. , Pynda M. , Pudiak V. , and Krupei V. , Quantity and Diameter of Dentinal Tubules of Human Teeth and Teeth of Experimental Animals According to Scanning Electron Microscopy Data, Regulatory Mechanisms in Biosystems. (2023) 14, no. 4, 609–616, 10.15421/022388.

